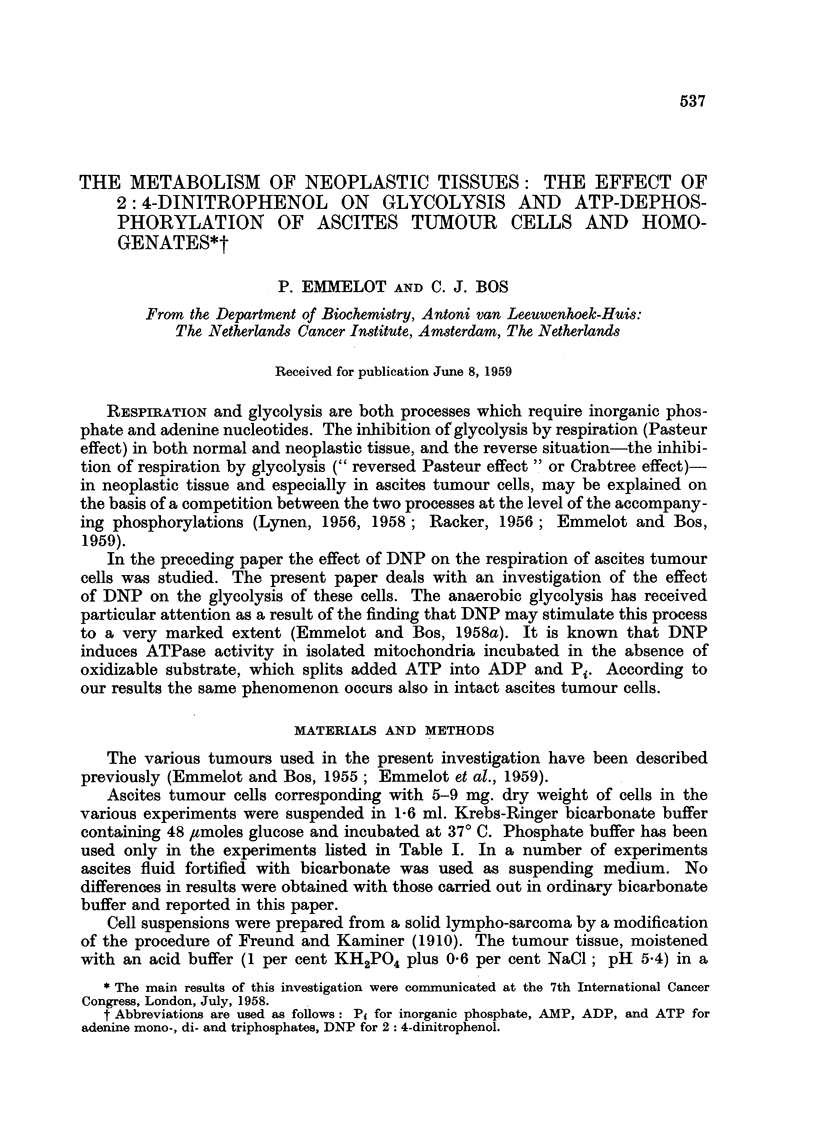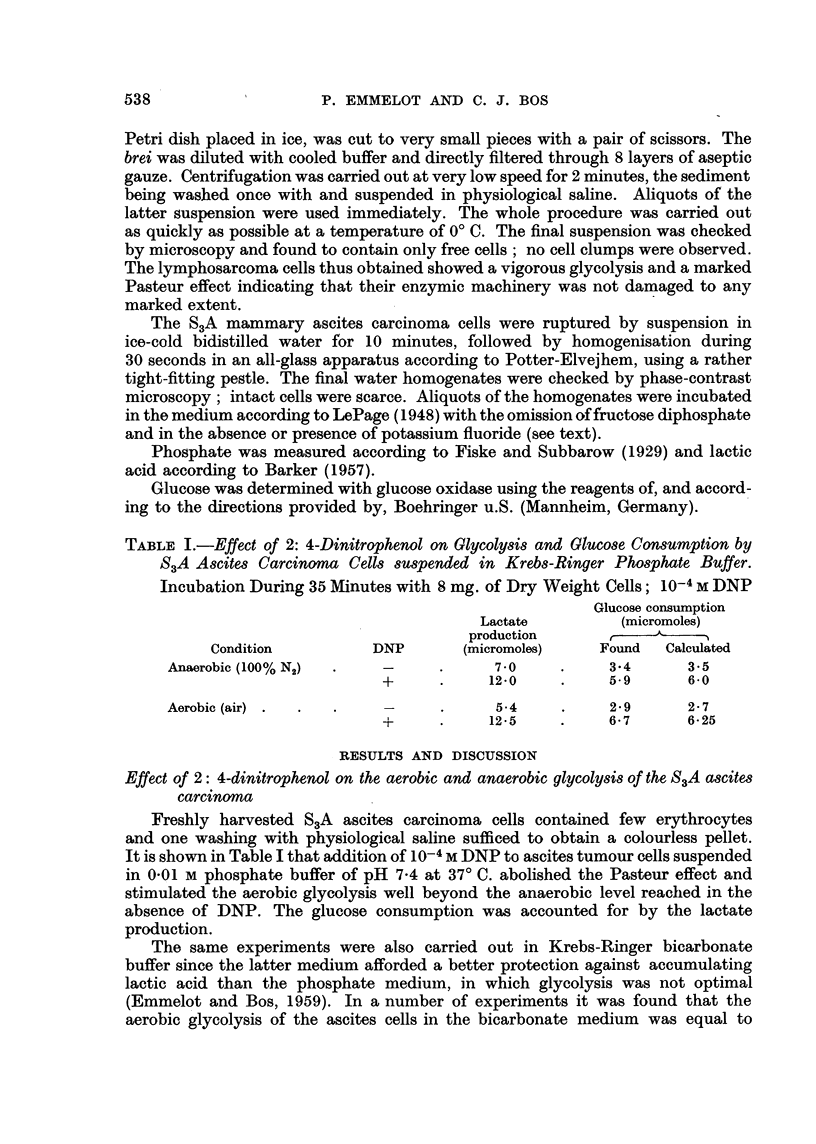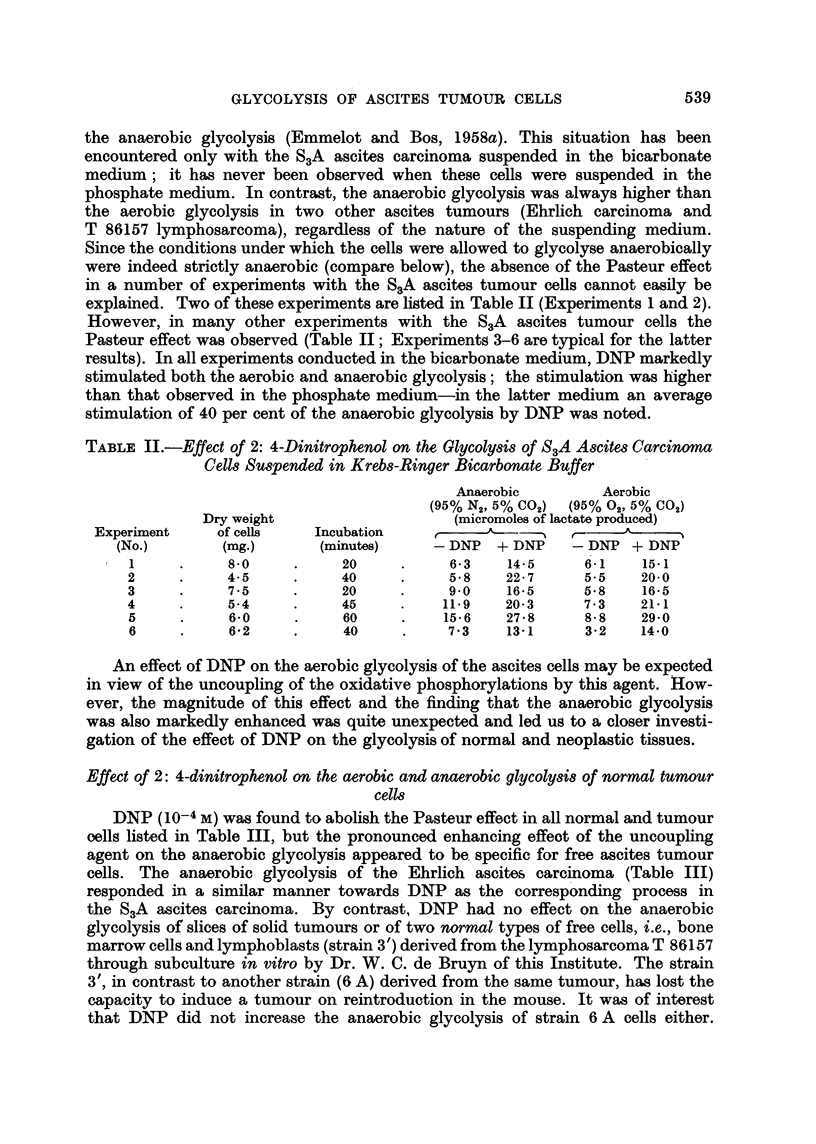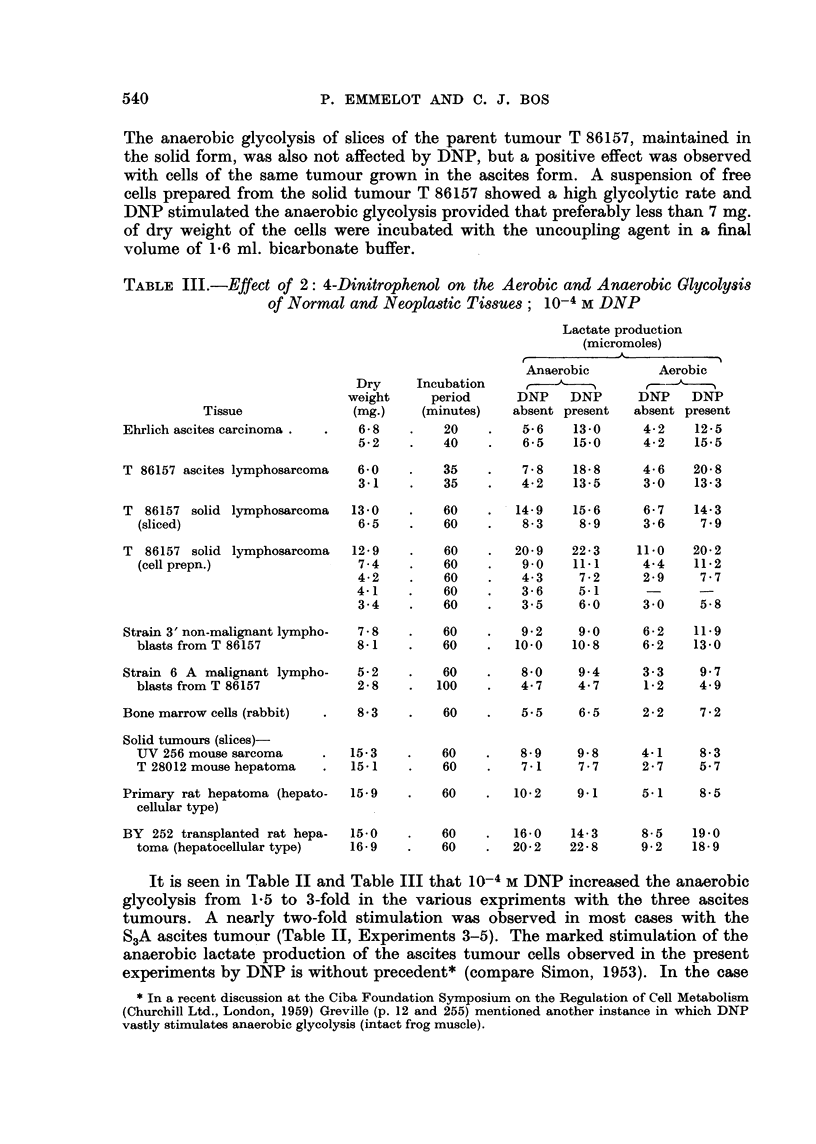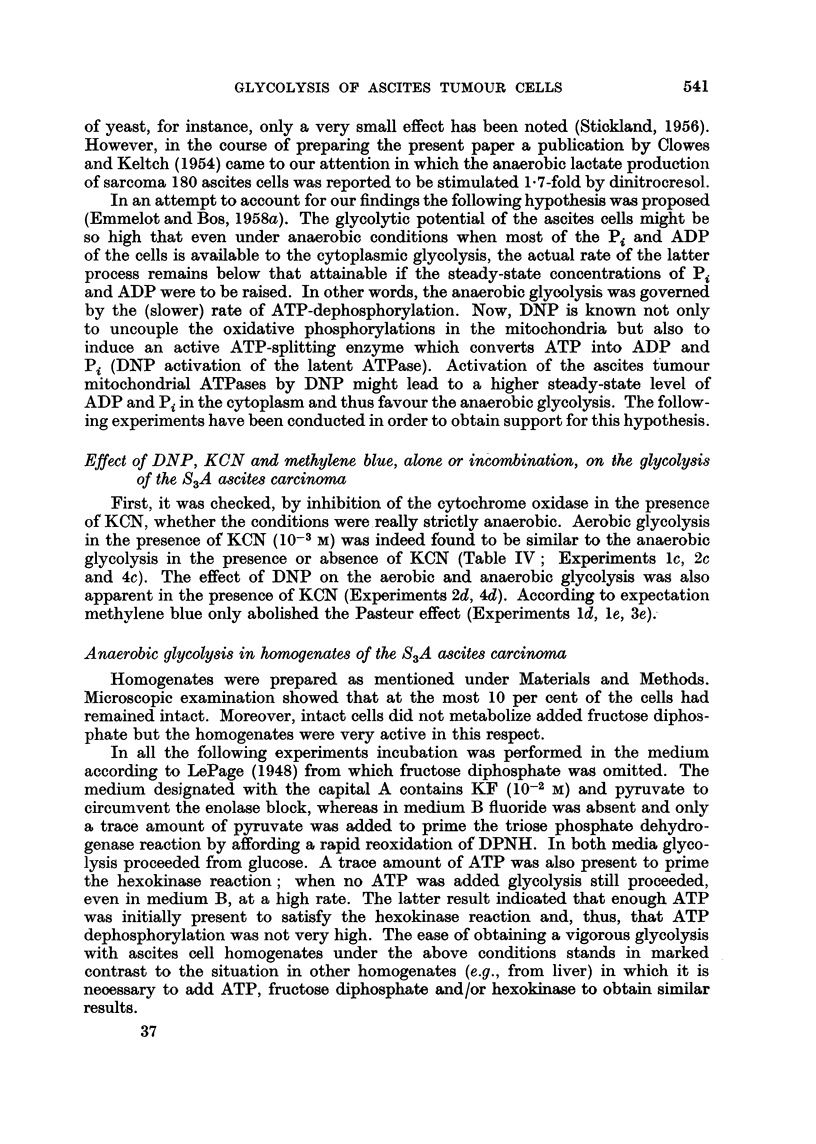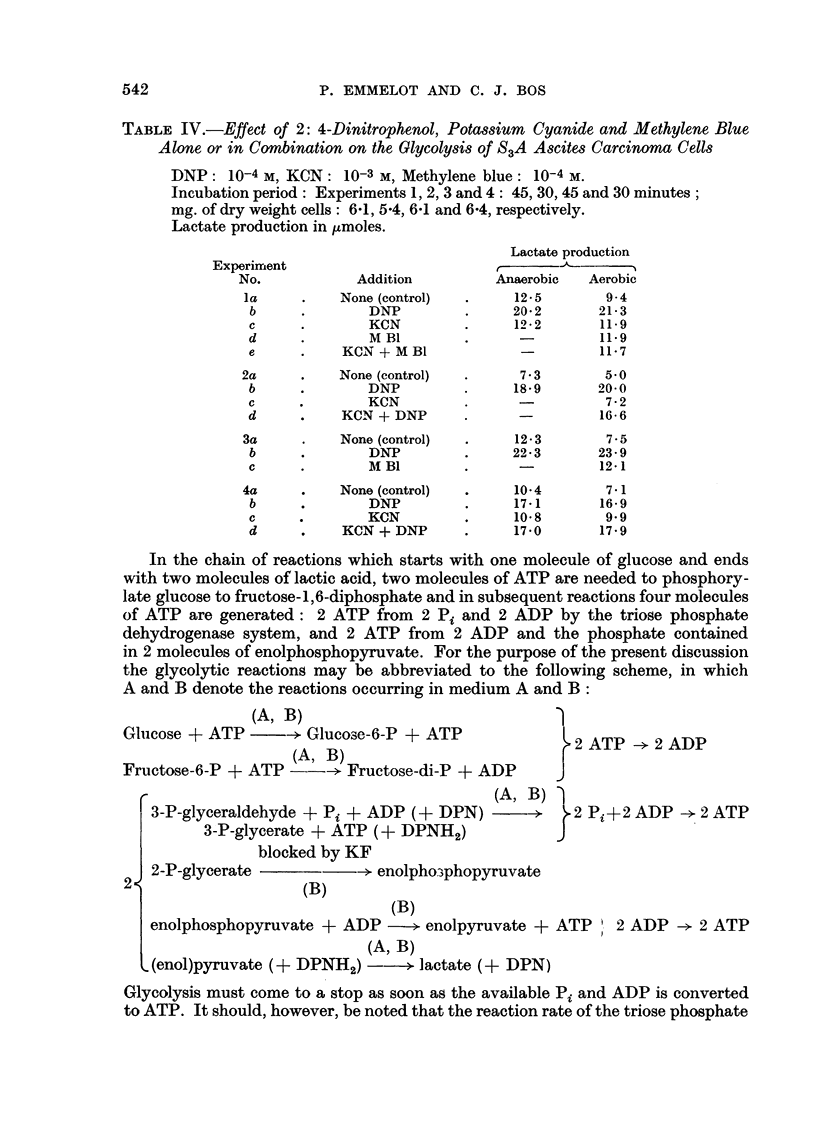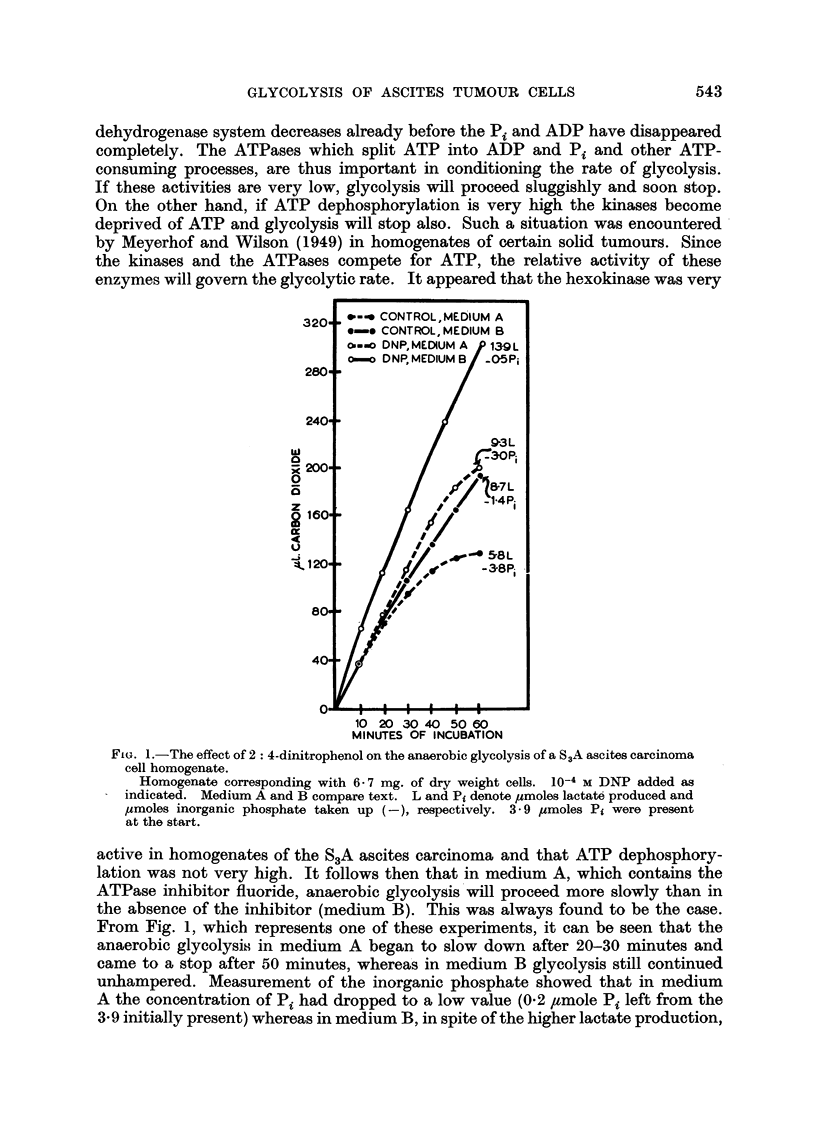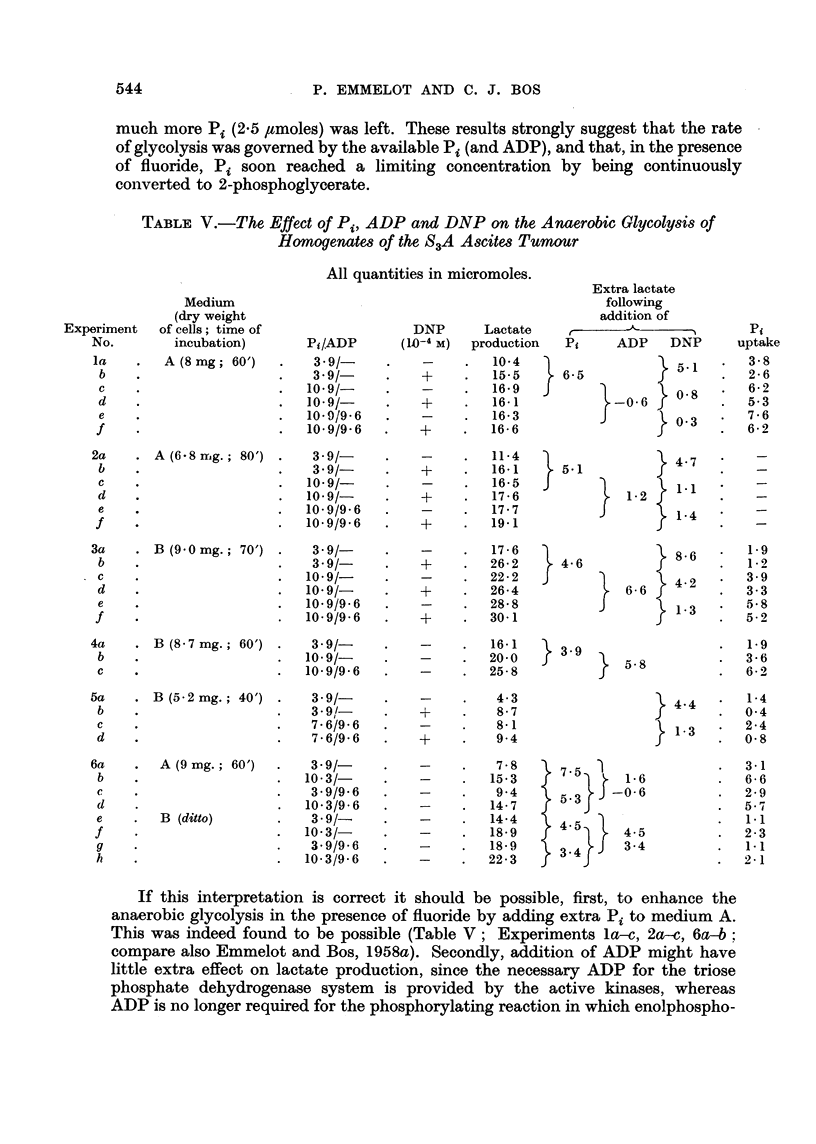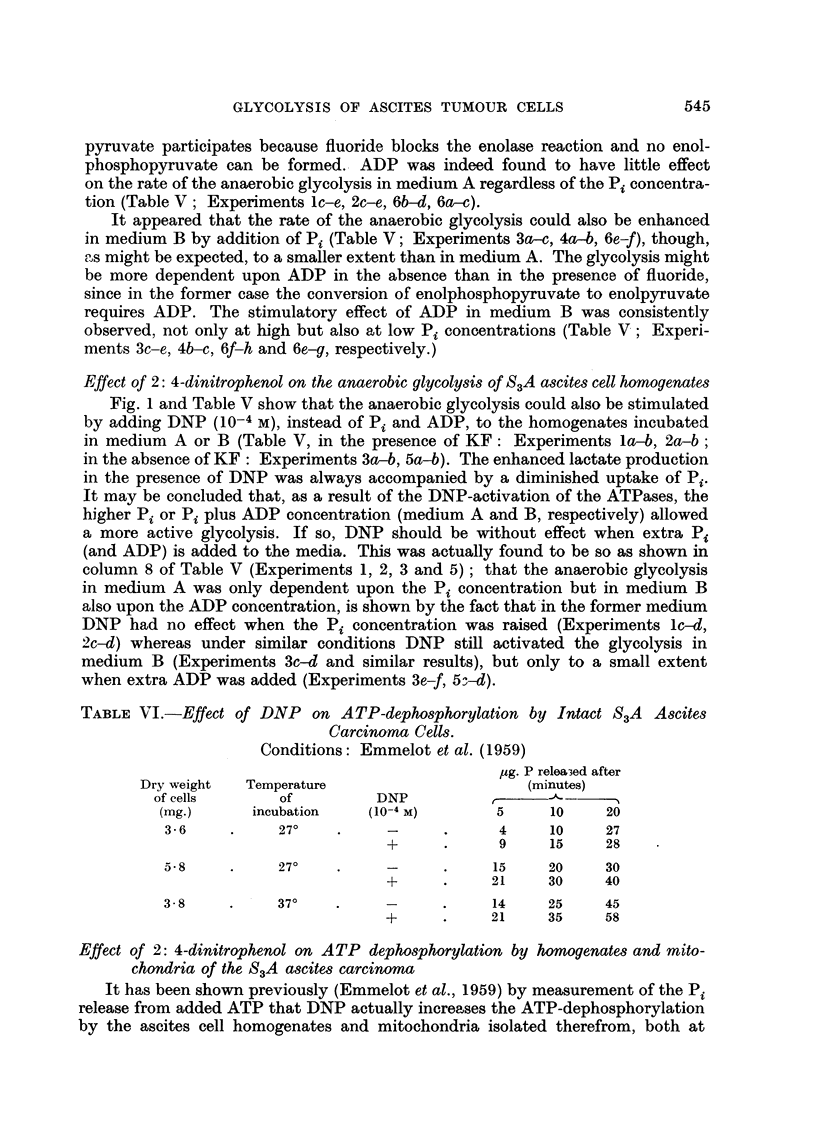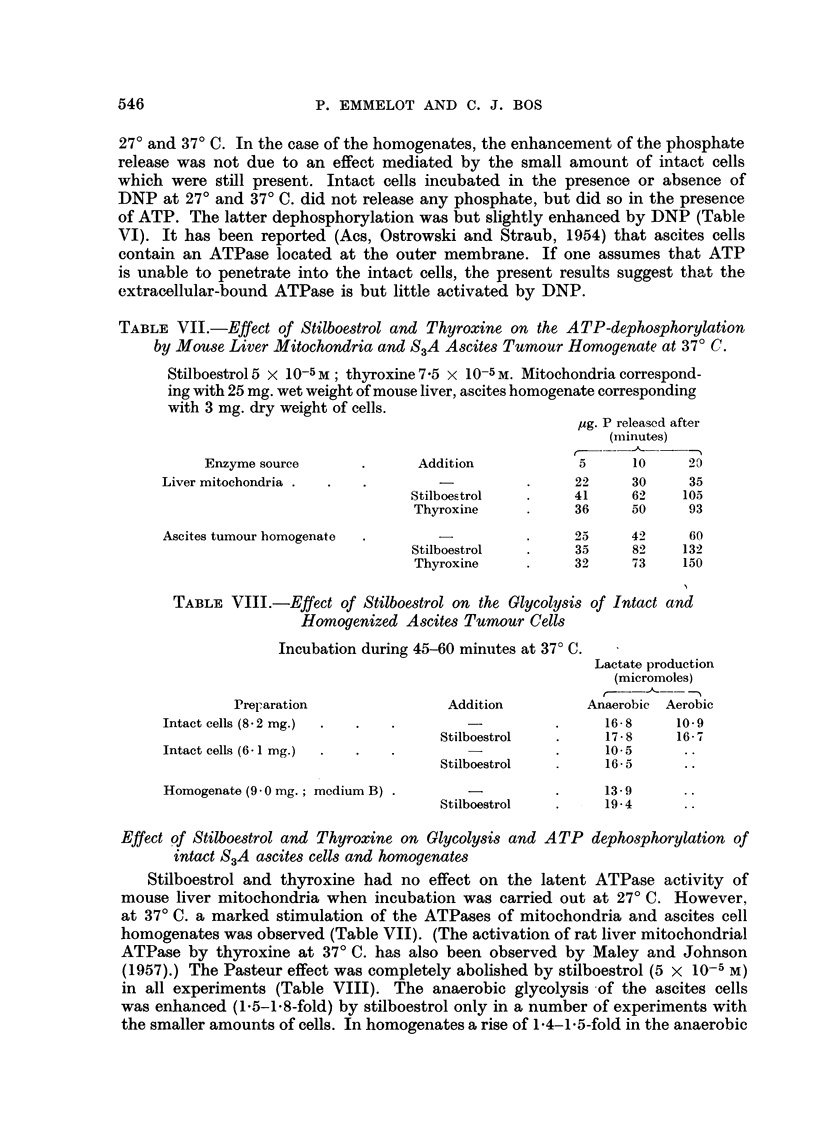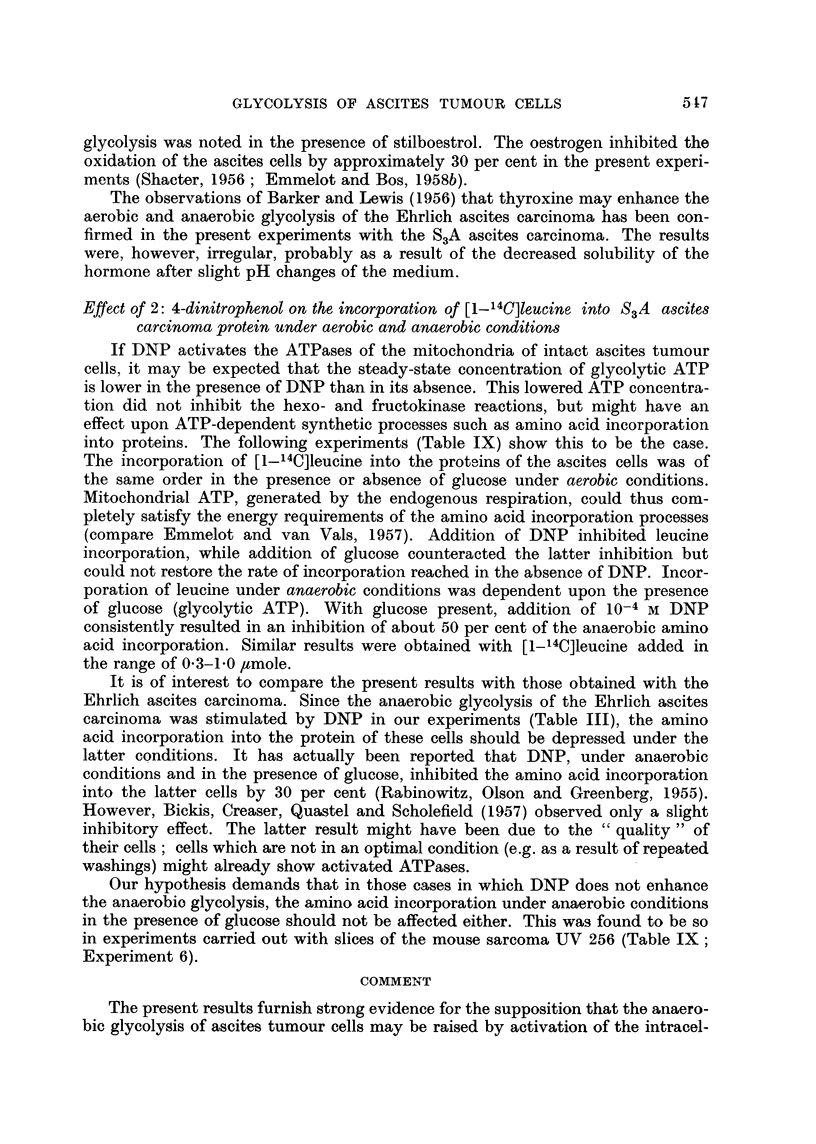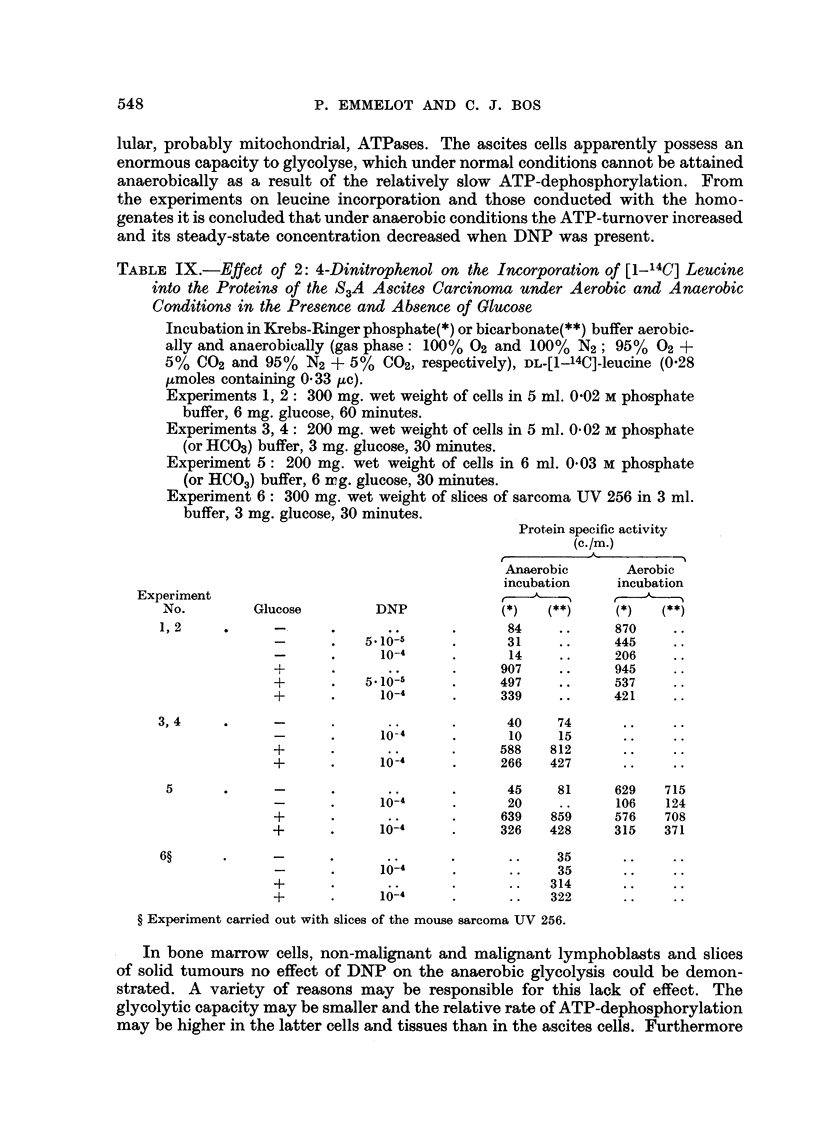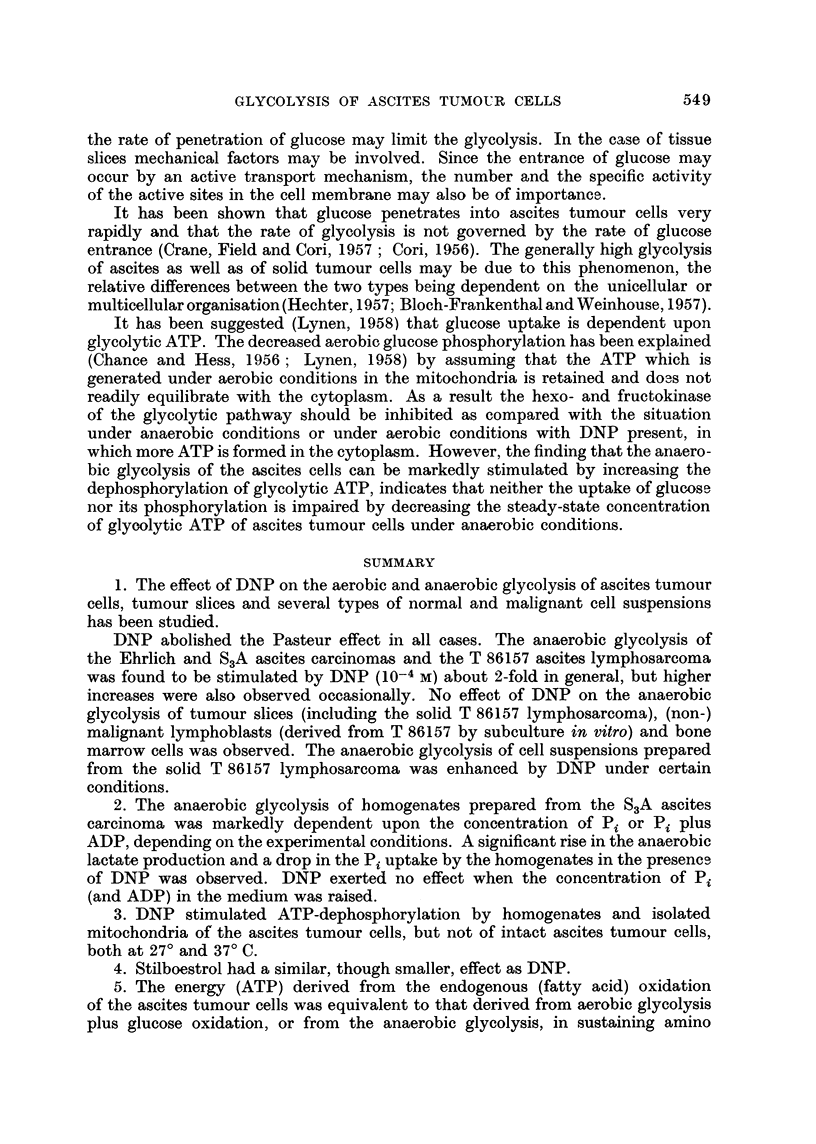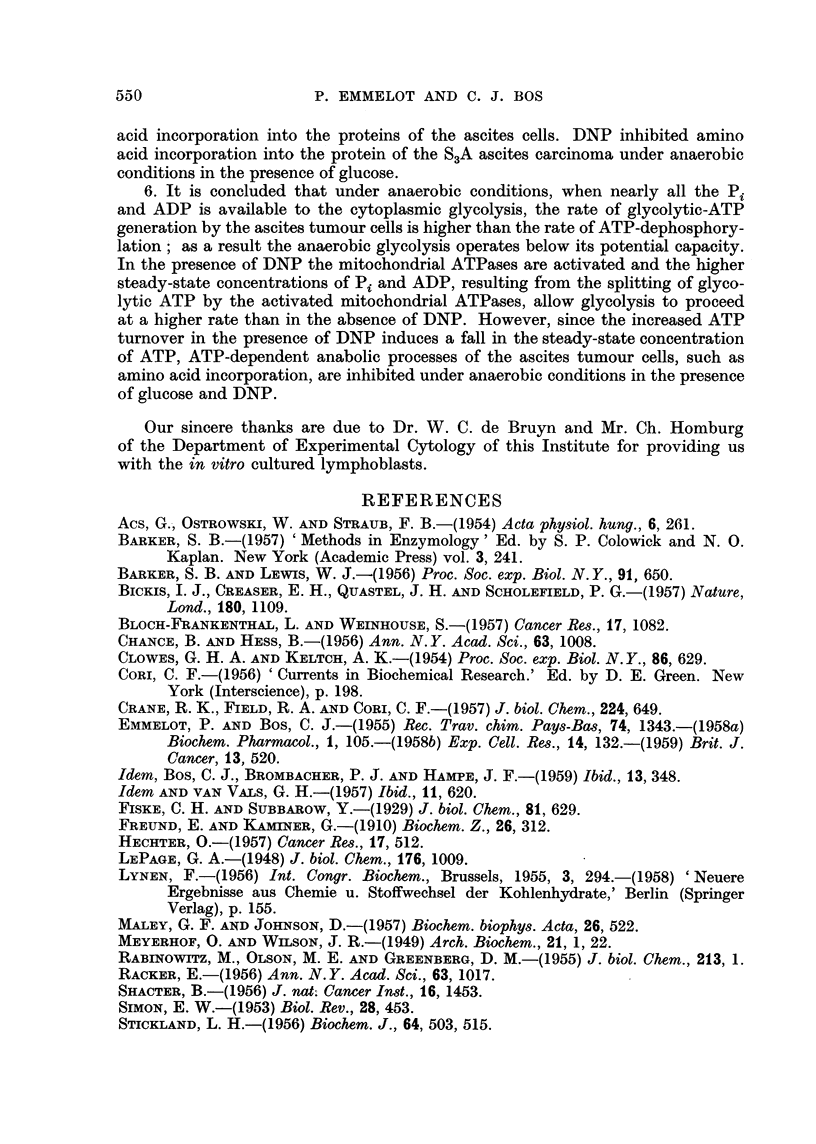# The Metabolism of Neoplastic Tissues: The Effect of 2:4-Dinitrophenol on Glycolysis and ATP-Dephosphorylation of Ascites Tumour Cells and Homogenates*,†

**DOI:** 10.1038/bjc.1959.59

**Published:** 1959-09

**Authors:** P. Emmelot, C. J. Bos


### THE METABOLISM OF NEOPLASTIC TISSUES: THE EFFECT OF 2:4-DINITROPHENOL ON GLYCOLYSIS AND ATP-DEPHOSPHORYLATION OF ASCITES TUMOUR CELLS AND HOMOGENATES*†

P. EMMELOT AND C. J. BOS

*From the Department of Biochemistry, Antoni van Leeuwenhoek-Huis: The Netherlands Cancer Institute, Amsterdam, The Netherlands*

Received for publication June 8, 1959

RESPIRATION and glycolysis are both processes which require inorganic phosphate and adenine nucleotides. The inhibition of glycolysis by respiration (Pasteur effect) in both normal and neoplastic tissue, and the reverse situation—the inhibition of respiration by glycolysis (" reversed Pasteur effect " or Crabtree effect)—in neoplastic tissue and especially in ascites tumour cells, may be explained on the basis of a competition between the two processes at the level of the accompanying phosphorylations (Lynen, 1956, 1958; Racker, 1956; Emmelot and Bos, 1959).

In the preceding paper the effect of DNP on the respiration of ascites tumour cells was studied. The present paper deals with an investigation of the effect of DNP on the glycolysis of these cells. The anaerobic glycolysis has received particular attention as a result of the finding that DNP may stimulate this process to a very marked extent (Emmelot and Bos, 1958*a*). It is known that DNP induces ATPase activity in isolated mitochondria incubated in the absence of oxidizable substrate, which splits added ATP into ADP and $P_i$. According to our results the same phenomenon occurs also in intact ascites tumour cells.

### MATERIALS AND METHODS

The various tumours used in the present investigation have been described previously (Emmelot and Bos, 1955; Emmelot *et al.*, 1959).

Ascites tumour cells corresponding with 5–9 mg. dry weight of cells in the various experiments were suspended in 1·6 ml. Krebs-Ringer bicarbonate buffer containing 48 $\mu$moles glucose and incubated at 37° C. Phosphate buffer has been used only in the experiments listed in Table I. In a number of experiments ascites fluid fortified with bicarbonate was used as suspending medium. No differences in results were obtained with those carried out in ordinary bicarbonate buffer and reported in this paper.

Cell suspensions were prepared from a solid lympho-sarcoma by a modification of the procedure of Freund and Kaminer (1910). The tumour tissue, moistened with an acid buffer (1 per cent $KH_2PO_4$ plus 0·6 per cent NaCl; pH 5·4) in a

* The main results of this investigation were communicated at the 7th International Cancer Congress, London, July, 1958.

† Abbreviations are used as follows: $P_i$ for inorganic phosphate, AMP, ADP, and ATP for adenine mono-, di- and triphosphates, DNP for 2 : 4-dinitrophenol.

Petri dish placed in ice, was cut to very small pieces with a pair of scissors. The *brei* was diluted with cooled buffer and directly filtered through 8 layers of aseptic gauze. Centrifugation was carried out at very low speed for 2 minutes, the sediment being washed once with and suspended in physiological saline. Aliquots of the latter suspension were used immediately. The whole procedure was carried out as quickly as possible at a temperature of 0° C. The final suspension was checked by microscopy and found to contain only free cells ; no cell clumps were observed. The lymphosarcoma cells thus obtained showed a vigorous glycolysis and a marked Pasteur effect indicating that their enzymic machinery was not damaged to any marked extent.

The $S_3A$ mammary ascites carcinoma cells were ruptured by suspension in ice-cold bidistilled water for 10 minutes, followed by homogenisation during 30 seconds in an all-glass apparatus according to Potter-Elvejhem, using a rather tight-fitting pestle. The final water homogenates were checked by phase-contrast microscopy ; intact cells were scarce. Aliquots of the homogenates were incubated in the medium according to LePage (1948) with the omission of fructose diphosphate and in the absence or presence of potassium fluoride (see text).

Phosphate was measured according to Fiske and Subbarow (1929) and lactic acid according to Barker (1957).

Glucose was determined with glucose oxidase using the reagents of, and according to the directions provided by, Boehringer u.S. (Mannheim, Germany).

TABLE I.—*Effect of* 2: 4-*Dinitrophenol on Glycolysis and Glucose Consumption by $S_3A$ Ascites Carcinoma Cells suspended in Krebs-Ringer Phosphate Buffer.* Incubation During 35 Minutes with 8 mg. of Dry Weight Cells; $10^{-4}$ M DNP

| Condition | DNP | Lactate production (micromoles) | Glucose consumption (micromoles) Found | Calculated |
|---|---|---|---|---|
| Anaerobic (100% $N_2$) | — | 7·0 | 3·4 | 3·5 |
| | + | 12·0 | 5·9 | 6·0 |
| Aerobic (air) | — | 5·4 | 2·9 | 2·7 |
| | + | 12·5 | 6·7 | 6·25 |

### RESULTS AND DISCUSSION

### *Effect of* 2: 4-*dinitrophenol on the aerobic and anaerobic glycolysis of the $S_3A$ ascites carcinoma*

Freshly harvested $S_3A$ ascites carcinoma cells contained few erythrocytes and one washing with physiological saline sufficed to obtain a colourless pellet. It is shown in Table I that addition of $10^{-4}$ M DNP to ascites tumour cells suspended in 0·01 M phosphate buffer of pH 7·4 at 37° C. abolished the Pasteur effect and stimulated the aerobic glycolysis well beyond the anaerobic level reached in the absence of DNP. The glucose consumption was accounted for by the lactate production.

The same experiments were also carried out in Krebs-Ringer bicarbonate buffer since the latter medium afforded a better protection against accumulating lactic acid than the phosphate medium, in which glycolysis was not optimal (Emmelot and Bos, 1959). In a number of experiments it was found that the aerobic glycolysis of the ascites cells in the bicarbonate medium was equal to the anaerobic glycolysis (Emmelot and Bos, 1958*a*). This situation has been encountered only with the $S_3A$ ascites carcinoma suspended in the bicarbonate medium; it has never been observed when these cells were suspended in the phosphate medium. In contrast, the anaerobic glycolysis was always higher than the aerobic glycolysis in two other ascites tumours (Ehrlich carcinoma and T 86157 lymphosarcoma), regardless of the nature of the suspending medium. Since the conditions under which the cells were allowed to glycolyse anaerobically were indeed strictly anaerobic (compare below), the absence of the Pasteur effect in a number of experiments with the $S_3A$ ascites tumour cells cannot easily be explained. Two of these experiments are listed in Table II (Experiments 1 and 2). However, in many other experiments with the $S_3A$ ascites tumour cells the Pasteur effect was observed (Table II; Experiments 3–6 are typical for the latter results). In all experiments conducted in the bicarbonate medium, DNP markedly stimulated both the aerobic and anaerobic glycolysis; the stimulation was higher than that observed in the phosphate medium—in the latter medium an average stimulation of 40 per cent of the anaerobic glycolysis by DNP was noted.

TABLE II.—*Effect of 2: 4-Dinitrophenol on the Glycolysis of $S_3A$ Ascites Carcinoma Cells Suspended in Krebs-Ringer Bicarbonate Buffer*

| Experiment (No.) | Dry weight of cells (mg.) | Incubation (minutes) | Anaerobic (95% $N_2$, 5% $CO_2$) (micromoles of lactate produced) | | Aerobic (95% $O_2$, 5% $CO_2$) (micromoles of lactate produced) | |
|---|---|---|---|---|---|---|
| | | | − DNP | + DNP | − DNP | + DNP |
| 1 | 8·0 | 20 | 6·3 | 14·5 | 6·1 | 15·1 |
| 2 | 4·5 | 40 | 5·8 | 22·7 | 5·5 | 20·0 |
| 3 | 7·5 | 20 | 9·0 | 16·5 | 5·8 | 16·5 |
| 4 | 5·4 | 45 | 11·9 | 20·3 | 7·3 | 21·1 |
| 5 | 6·0 | 60 | 15·6 | 27·8 | 8·8 | 29·0 |
| 6 | 6·2 | 40 | 7·3 | 13·1 | 3·2 | 14·0 |

An effect of DNP on the aerobic glycolysis of the ascites cells may be expected in view of the uncoupling of the oxidative phosphorylations by this agent. However, the magnitude of this effect and the finding that the anaerobic glycolysis was also markedly enhanced was quite unexpected and led us to a closer investigation of the effect of DNP on the glycolysis of normal and neoplastic tissues.

*Effect of 2: 4-dinitrophenol on the aerobic and anaerobic glycolysis of normal tumour cells*

DNP ($10^{-4}$ M) was found to abolish the Pasteur effect in all normal and tumour cells listed in Table III, but the pronounced enhancing effect of the uncoupling agent on the anaerobic glycolysis appeared to be specific for free ascites tumour cells. The anaerobic glycolysis of the Ehrlich ascites carcinoma (Table III) responded in a similar manner towards DNP as the corresponding process in the $S_3A$ ascites carcinoma. By contrast, DNP had no effect on the anaerobic glycolysis of slices of solid tumours or of two *normal* types of free cells, *i.e.*, bone marrow cells and lymphoblasts (strain 3′) derived from the lymphosarcoma T 86157 through subculture *in vitro* by Dr. W. C. de Bruyn of this Institute. The strain 3′, in contrast to another strain (6 A) derived from the same tumour, has lost the capacity to induce a tumour on reintroduction in the mouse. It was of interest that DNP did not increase the anaerobic glycolysis of strain 6 A cells either.

The anaerobic glycolysis of slices of the parent tumour T 86157, maintained in the solid form, was also not affected by DNP, but a positive effect was observed with cells of the same tumour grown in the ascites form. A suspension of free cells prepared from the solid tumour T 86157 showed a high glycolytic rate and DNP stimulated the anaerobic glycolysis provided that preferably less than 7 mg. of dry weight of the cells were incubated with the uncoupling agent in a final volume of 1·6 ml. bicarbonate buffer.

TABLE III.—*Effect of 2 : 4-Dinitrophenol on the Aerobic and Anaerobic Glycolysis of Normal and Neoplastic Tissues* ; $10^{-4}$ M *DNP*

| | | | Lactate production (micromoles) | | | |
|---|---|---|---|---|---|---|
| | | | Anaerobic | | Aerobic | |
| Tissue | Dry weight (mg.) | Incubation period (minutes) | DNP absent | DNP present | DNP absent | DNP present |
| Ehrlich ascites carcinoma | 6·8 | 20 | 5·6 | 13·0 | 4·2 | 12·5 |
| | 5·2 | 40 | 6·5 | 15·0 | 4·2 | 15·5 |
| T 86157 ascites lymphosarcoma | 6·0 | 35 | 7·8 | 18·8 | 4·6 | 20·8 |
| | 3·1 | 35 | 4·2 | 13·5 | 3·0 | 13·3 |
| T 86157 solid lymphosarcoma (sliced) | 13·0 | 60 | 14·9 | 15·6 | 6·7 | 14·3 |
| | 6·5 | 60 | 8·3 | 8·9 | 3·6 | 7·9 |
| T 86157 solid lymphosarcoma (cell prepn.) | 12·9 | 60 | 20·9 | 22·3 | 11·0 | 20·2 |
| | 7·4 | 60 | 9·0 | 11·1 | 4·4 | 11·2 |
| | 4·2 | 60 | 4·3 | 7·2 | 2·9 | 7·7 |
| | 4·1 | 60 | 3·6 | 5·1 | — | — |
| | 3·4 | 60 | 3·5 | 6·0 | 3·0 | 5·8 |
| Strain 3′ non-malignant lymphoblasts from T 86157 | 7·8 | 60 | 9·2 | 9·0 | 6·2 | 11·9 |
| | 8·1 | 60 | 10·0 | 10·8 | 6·2 | 13·0 |
| Strain 6 A malignant lymphoblasts from T 86157 | 5·2 | 60 | 8·0 | 9·4 | 3·3 | 9·7 |
| | 2·8 | 100 | 4·7 | 4·7 | 1·2 | 4·9 |
| Bone marrow cells (rabbit) | 8·3 | 60 | 5·5 | 6·5 | 2·2 | 7·2 |
| Solid tumours (slices)— | | | | | | |
| UV 256 mouse sarcoma | 15·3 | 60 | 8·9 | 9·8 | 4·1 | 8·3 |
| T 28012 mouse hepatoma | 15·1 | 60 | 7·1 | 7·7 | 2·7 | 5·7 |
| Primary rat hepatoma (hepatocellular type) | 15·9 | 60 | 10·2 | 9·1 | 5·1 | 8·5 |
| BY 252 transplanted rat hepatoma (hepatocellular type) | 15·0 | 60 | 16·0 | 14·3 | 8·5 | 19·0 |
| | 16·9 | 60 | 20·2 | 22·8 | 9·2 | 18·9 |

It is seen in Table II and Table III that $10^{-4}$ M DNP increased the anaerobic glycolysis from 1·5 to 3-fold in the various expriments with the three ascites tumours. A nearly two-fold stimulation was observed in most cases with the $S_3A$ ascites tumour (Table II, Experiments 3–5). The marked stimulation of the anaerobic lactate production of the ascites tumour cells observed in the present experiments by DNP is without precedent* (compare Simon, 1953). In the case

* In a recent discussion at the Ciba Foundation Symposium on the Regulation of Cell Metabolism (Churchill Ltd., London, 1959) Greville (p. 12 and 255) mentioned another instance in which DNP vastly stimulates anaerobic glycolysis (intact frog muscle).

of yeast, for instance, only a very small effect has been noted (Stickland, 1956). However, in the course of preparing the present paper a publication by Clowes and Keltch (1954) came to our attention in which the anaerobic lactate production of sarcoma 180 ascites cells was reported to be stimulated 1·7-fold by dinitrocresol.

In an attempt to account for our findings the following hypothesis was proposed (Emmelot and Bos, 1958*a*). The glycolytic potential of the ascites cells might be so high that even under anaerobic conditions when most of the $P_i$ and ADP of the cells is available to the cytoplasmic glycolysis, the actual rate of the latter process remains below that attainable if the steady-state concentrations of $P_i$ and ADP were to be raised. In other words, the anaerobic glycolysis was governed by the (slower) rate of ATP-dephosphorylation. Now, DNP is known not only to uncouple the oxidative phosphorylations in the mitochondria but also to induce an active ATP-splitting enzyme which converts ATP into ADP and $P_i$ (DNP activation of the latent ATPase). Activation of the ascites tumour mitochondrial ATPases by DNP might lead to a higher steady-state level of ADP and $P_i$ in the cytoplasm and thus favour the anaerobic glycolysis. The following experiments have been conducted in order to obtain support for this hypothesis.

*Effect of DNP, KCN and methylene blue, alone or incombination, on the glycolysis of the $S_3A$ ascites carcinoma*

First, it was checked, by inhibition of the cytochrome oxidase in the presence of KCN, whether the conditions were really strictly anaerobic. Aerobic glycolysis in the presence of KCN ($10^{-3}$ M) was indeed found to be similar to the anaerobic glycolysis in the presence or absence of KCN (Table IV; Experiments 1*c*, 2*c* and 4*c*). The effect of DNP on the aerobic and anaerobic glycolysis was also apparent in the presence of KCN (Experiments 2*d*, 4*d*). According to expectation methylene blue only abolished the Pasteur effect (Experiments 1*d*, 1*e*, 3*e*).

*Anaerobic glycolysis in homogenates of the $S_3A$ ascites carcinoma*

Homogenates were prepared as mentioned under Materials and Methods. Microscopic examination showed that at the most 10 per cent of the cells had remained intact. Moreover, intact cells did not metabolize added fructose diphosphate but the homogenates were very active in this respect.

In all the following experiments incubation was performed in the medium according to LePage (1948) from which fructose diphosphate was omitted. The medium designated with the capital A contains KF ($10^{-2}$ M) and pyruvate to circumvent the enolase block, whereas in medium B fluoride was absent and only a trace amount of pyruvate was added to prime the triose phosphate dehydrogenase reaction by affording a rapid reoxidation of DPNH. In both media glycolysis proceeded from glucose. A trace amount of ATP was also present to prime the hexokinase reaction; when no ATP was added glycolysis still proceeded, even in medium B, at a high rate. The latter result indicated that enough ATP was initially present to satisfy the hexokinase reaction and, thus, that ATP dephosphorylation was not very high. The ease of obtaining a vigorous glycolysis with ascites cell homogenates under the above conditions stands in marked contrast to the situation in other homogenates (*e.g.*, from liver) in which it is necessary to add ATP, fructose diphosphate and/or hexokinase to obtain similar results.

TABLE IV.—*Effect of 2: 4-Dinitrophenol, Potassium Cyanide and Methylene Blue Alone or in Combination on the Glycolysis of $S_3A$ Ascites Carcinoma Cells*

DNP: $10^{-4}$ M, KCN: $10^{-3}$ M, Methylene blue: $10^{-4}$ M.
Incubation period: Experiments 1, 2, 3 and 4: 45, 30, 45 and 30 minutes; mg. of dry weight cells: 6·1, 5·4, 6·1 and 6·4, respectively.
Lactate production in $\mu$moles.

| Experiment No. | Addition | Lactate production Anaerobic | Aerobic |
|---|---|---|---|
| 1*a* | None (control) | 12·5 | 9·4 |
| *b* | DNP | 20·2 | 21·3 |
| *c* | KCN | 12·2 | 11·9 |
| *d* | M Bl | — | 11·9 |
| *e* | KCN + M Bl | — | 11·7 |
| 2*a* | None (control) | 7·3 | 5·0 |
| *b* | DNP | 18·9 | 20·0 |
| *c* | KCN | — | 7·2 |
| *d* | KCN + DNP | — | 16·6 |
| 3*a* | None (control) | 12·3 | 7·5 |
| *b* | DNP | 22·3 | 23·9 |
| *c* | M Bl | — | 12·1 |
| 4*a* | None (control) | 10·4 | 7·1 |
| *b* | DNP | 17·1 | 16·9 |
| *c* | KCN | 10·8 | 9·9 |
| *d* | KCN + DNP | 17·0 | 17·9 |

In the chain of reactions which starts with one molecule of glucose and ends with two molecules of lactic acid, two molecules of ATP are needed to phosphorylate glucose to fructose-1,6-diphosphate and in subsequent reactions four molecules of ATP are generated: 2 ATP from 2 $P_i$ and 2 ADP by the triose phosphate dehydrogenase system, and 2 ATP from 2 ADP and the phosphate contained in 2 molecules of enolphosphopyruvate. For the purpose of the present discussion the glycolytic reactions may be abbreviated to the following scheme, in which A and B denote the reactions occurring in medium A and B:

Glucose + ATP —(A, B)→ Glucose-6-P + ATP

Fructose-6-P + ATP —(A, B)→ Fructose-di-P + ADP

} 2 ATP → 2 ADP

2 {

3-P-glyceraldehyde + $P_i$ + ADP (+ DPN) —(A, B)→ 3-P-glycerate + ATP (+ $DPNH_2$) } 2 $P_i$+2 ADP → 2 ATP

2-P-glycerate —(B), blocked by KF→ enolphosphopyruvate enolphosphopyruvate + ADP —(B)→ enolpyruvate + ATP } 2 ADP → 2 ATP

(enol)pyruvate (+ $DPNH_2$) —(A, B)→ lactate (+ DPN)

}

Glycolysis must come to a stop as soon as the available $P_i$ and ADP is converted to ATP. It should, however, be noted that the reaction rate of the triose phosphate dehydrogenase system decreases already before the $P_i$ and ADP have disappeared completely. The ATPases which split ATP into ADP and $P_i$ and other ATP-consuming processes, are thus important in conditioning the rate of glycolysis. If these activities are very low, glycolysis will proceed sluggishly and soon stop. On the other hand, if ATP dephosphorylation is very high the kinases become deprived of ATP and glycolysis will stop also. Such a situation was encountered by Meyerhof and Wilson (1949) in homogenates of certain solid tumours. Since the kinases and the ATPases compete for ATP, the relative activity of these enzymes will govern the glycolytic rate. It appeared that the hexokinase was very

FIG. 1.—The effect of 2 : 4-dinitrophenol on the anaerobic glycolysis of a $S_3A$ ascites carcinoma cell homogenate.

Homogenate corresponding with 6·7 mg. of dry weight cells. $10^{-4}$ M DNP added as indicated. Medium A and B compare text. L and $P_i$ denote μmoles lactate produced and μmoles inorganic phosphate taken up (—), respectively. 3·9 μmoles $P_i$ were present at the start.

active in homogenates of the $S_3A$ ascites carcinoma and that ATP dephosphorylation was not very high. It follows then that in medium A, which contains the ATPase inhibitor fluoride, anaerobic glycolysis will proceed more slowly than in the absence of the inhibitor (medium B). This was always found to be the case. From Fig. 1, which represents one of these experiments, it can be seen that the anaerobic glycolysis in medium A began to slow down after 20–30 minutes and came to a stop after 50 minutes, whereas in medium B glycolysis still continued unhampered. Measurement of the inorganic phosphate showed that in medium A the concentration of $P_i$ had dropped to a low value (0·2 μmole $P_i$ left from the 3·9 initially present) whereas in medium B, in spite of the higher lactate production, much more $P_i$ (2·5 μmoles) was left. These results strongly suggest that the rate of glycolysis was governed by the available $P_i$ (and ADP), and that, in the presence of fluoride, $P_i$ soon reached a limiting concentration by being continuously converted to 2-phosphoglycerate.

TABLE V.—*The Effect of $P_i$, ADP and DNP on the Anaerobic Glycolysis of Homogenates of the $S_3A$ Ascites Tumour*

All quantities in micromoles.

| Experiment No. | Medium (dry weight of cells; time of incubation) | $P_i$/ADP | DNP ($10^{-4}$ M) | Lactate production | Extra lactate following addition of $P_i$ | ADP | DNP | $P_i$ uptake |
|---|---|---|---|---|---|---|---|---|
| 1*a* | A (8 mg; 60′) | 3·9/— | — | 10·4 | | | 5·1 | 3·8 |
| *b* | | 3·9/— | + | 15·5 | 6·5 | | | 2·6 |
| *c* | | 10·9/— | — | 16·9 | | | 0·8 | 6·2 |
| *d* | | 10·9/— | + | 16·1 | | −0·6 | | 5·3 |
| *e* | | 10·9/9·6 | — | 16·3 | | | 0·3 | 7·6 |
| *f* | | 10·9/9·6 | + | 16·6 | | | | 6·2 |
| 2*a* | A (6·8 mg.; 80′) | 3·9/— | — | 11·4 | | | 4·7 | — |
| *b* | | 3·9/— | + | 16·1 | 5·1 | | | — |
| *c* | | 10·9/— | — | 16·5 | | | 1·1 | — |
| *d* | | 10·9/— | + | 17·6 | | 1·2 | | — |
| *e* | | 10·9/9·6 | — | 17·7 | | | 1·4 | — |
| *f* | | 10·9/9·6 | + | 19·1 | | | | — |
| 3*a* | B (9·0 mg.; 70′) | 3·9/— | — | 17·6 | | | 8·6 | 1·9 |
| *b* | | 3·9/— | + | 26·2 | 4·6 | | | 1·2 |
| *c* | | 10·9/— | — | 22·2 | | | 4·2 | 3·9 |
| *d* | | 10·9/— | + | 26·4 | | 6·6 | | 3·3 |
| *e* | | 10·9/9·6 | — | 28·8 | | | 1·3 | 5·8 |
| *f* | | 10·9/9·6 | + | 30·1 | | | | 5·2 |
| 4*a* | B (8·7 mg.; 60′) | 3·9/— | — | 16·1 | 3·9 | | | 1·9 |
| *b* | | 10·9/— | — | 20·0 | | 5·8 | | 3·6 |
| *c* | | 10·9/9·6 | — | 25·8 | | | | 6·2 |
| 5*a* | B (5·2 mg.; 40′) | 3·9/— | — | 4·3 | | | 4·4 | 1·4 |
| *b* | | 3·9/— | + | 8·7 | | | | 0·4 |
| *c* | | 7·6/9·6 | — | 8·1 | | | 1·3 | 2·4 |
| *d* | | 7·6/9·6 | + | 9·4 | | | | 0·8 |
| 6*a* | A (9 mg.; 60′) | 3·9/— | — | 7·8 | 7·5 | | | 3·1 |
| *b* | | 10·3/— | — | 15·3 | | 1·6 | | 6·6 |
| *c* | | 3·9/9·6 | — | 9·4 | | −0·6 | | 2·9 |
| *d* | | 10·3/9·6 | — | 14·7 | 5·3 | | | 5·7 |
| *e* | B (*ditto*) | 3·9/— | — | 14·4 | 4·5 | | | 1·1 |
| *f* | | 10·3/— | — | 18·9 | | 4·5 | | 2·3 |
| *g* | | 3·9/9·6 | — | 18·9 | | 3·4 | | 1·1 |
| *h* | | 10·3/9·6 | — | 22·3 | 3·4 | | | 2·1 |

If this interpretation is correct it should be possible, first, to enhance the anaerobic glycolysis in the presence of fluoride by adding extra $P_i$ to medium A. This was indeed found to be possible (Table V; Experiments 1*a–c*, 2*a–c*, 6*a–b*; compare also Emmelot and Bos, 1958*a*). Secondly, addition of ADP might have little extra effect on lactate production, since the necessary ADP for the triose phosphate dehydrogenase system is provided by the active kinases, whereas ADP is no longer required for the phosphorylating reaction in which enolphosphopyruvate participates because fluoride blocks the enolase reaction and no enolphosphopyruvate can be formed. ADP was indeed found to have little effect on the rate of the anaerobic glycolysis in medium A regardless of the $P_i$ concentration (Table V ; Experiments 1*c–e*, 2*c–e*, 6*b–d*, 6*a–c*).

It appeared that the rate of the anaerobic glycolysis could also be enhanced in medium B by addition of $P_i$ (Table V ; Experiments 3*a–c*, 4*a–b*, 6*e–f*), though, as might be expected, to a smaller extent than in medium A. The glycolysis might be more dependent upon ADP in the absence than in the presence of fluoride, since in the former case the conversion of enolphosphopyruvate to enolpyruvate requires ADP. The stimulatory effect of ADP in medium B was consistently observed, not only at high but also at low $P_i$ concentrations (Table V ; Experiments 3*c–e*, 4*b–c*, 6*f–h* and 6*e–g*, respectively.)

*Effect of 2: 4-dinitrophenol on the anaerobic glycolysis of $S_3A$ ascites cell homogenates*

Fig. 1 and Table V show that the anaerobic glycolysis could also be stimulated by adding DNP ($10^{-4}$ M), instead of $P_i$ and ADP, to the homogenates incubated in medium A or B (Table V, in the presence of KF : Experiments 1*a–b*, 2*a–b* ; in the absence of KF : Experiments 3*a–b*, 5*a–b*). The enhanced lactate production in the presence of DNP was always accompanied by a diminished uptake of $P_i$. It may be concluded that, as a result of the DNP-activation of the ATPases, the higher $P_i$ or $P_i$ plus ADP concentration (medium A and B, respectively) allowed a more active glycolysis. If so, DNP should be without effect when extra $P_i$ (and ADP) is added to the media. This was actually found to be so as shown in column 8 of Table V (Experiments 1, 2, 3 and 5) ; that the anaerobic glycolysis in medium A was only dependent upon the $P_i$ concentration but in medium B also upon the ADP concentration, is shown by the fact that in the former medium DNP had no effect when the $P_i$ concentration was raised (Experiments 1*c–d*, 2*c–d*) whereas under similar conditions DNP still activated the glycolysis in medium B (Experiments 3*c–d* and similar results), but only to a small extent when extra ADP was added (Experiments 3*e–f*, 5*c–d*).

TABLE VI.—*Effect of DNP on ATP-dephosphorylation by Intact $S_3A$ Ascites Carcinoma Cells.*

Conditions: Emmelot *et al.* (1959)

| Dry weight of cells (mg.) | Temperature of incubation | DNP ($10^{-4}$ M) | μg. P released after (minutes) 5 | 10 | 20 |
|---|---|---|---|---|---|
| 3·6 | 27° | — | 4 | 10 | 27 |
| | | + | 9 | 15 | 28 |
| 5·8 | 27° | — | 15 | 20 | 30 |
| | | + | 21 | 30 | 40 |
| 3·8 | 37° | — | 14 | 25 | 45 |
| | | + | 21 | 35 | 58 |

*Effect of 2: 4-dinitrophenol on ATP dephosphorylation by homogenates and mitochondria of the $S_3A$ ascites carcinoma*

It has been shown previously (Emmelot *et al.*, 1959) by measurement of the $P_i$ release from added ATP that DNP actually increases the ATP-dephosphorylation by the ascites cell homogenates and mitochondria isolated therefrom, both at

27° and 37° C. In the case of the homogenates, the enhancement of the phosphate release was not due to an effect mediated by the small amount of intact cells which were still present. Intact cells incubated in the presence or absence of DNP at 27° and 37° C. did not release any phosphate, but did so in the presence of ATP. The latter dephosphorylation was but slightly enhanced by DNP (Table VI). It has been reported (Acs, Ostrowski and Straub, 1954) that ascites cells contain an ATPase located at the outer membrane. If one assumes that ATP is unable to penetrate into the intact cells, the present results suggest that the extracellular-bound ATPase is but little activated by DNP.

TABLE VII.—*Effect of Stilboestrol and Thyroxine on the ATP-dephosphorylation by Mouse Liver Mitochondria and $S_3A$ Ascites Tumour Homogenate at 37° C.*

Stilboestrol $5 \times 10^{-5}$ M; thyroxine $7 \cdot 5 \times 10^{-5}$ M. Mitochondria corresponding with 25 mg. wet weight of mouse liver, ascites homogenate corresponding with 3 mg. dry weight of cells.

| Enzyme source | Addition | μg. P released after (minutes) 5 | 10 | 20 |
|---|---|---|---|---|
| Liver mitochondria | — | 22 | 30 | 35 |
| | Stilboestrol | 41 | 62 | 105 |
| | Thyroxine | 36 | 50 | 93 |
| Ascites tumour homogenate | — | 25 | 42 | 60 |
| | Stilboestrol | 35 | 82 | 132 |
| | Thyroxine | 32 | 73 | 150 |

TABLE VIII.—*Effect of Stilboestrol on the Glycolysis of Intact and Homogenized Ascites Tumour Cells*

Incubation during 45–60 minutes at 37° C.

| Preparation | Addition | Lactate production (micromoles) Anaerobic | Aerobic |
|---|---|---|---|
| Intact cells (8·2 mg.) | — | 16·8 | 10·9 |
| | Stilboestrol | 17·8 | 16·7 |
| Intact cells (6·1 mg.) | — | 10·5 | .. |
| | Stilboestrol | 16·5 | .. |
| Homogenate (9·0 mg.; medium B) | — | 13·9 | .. |
| | Stilboestrol | 19·4 | .. |

*Effect of Stilboestrol and Thyroxine on Glycolysis and ATP dephosphorylation of intact $S_3A$ ascites cells and homogenates*

Stilboestrol and thyroxine had no effect on the latent ATPase activity of mouse liver mitochondria when incubation was carried out at 27° C. However, at 37° C. a marked stimulation of the ATPases of mitochondria and ascites cell homogenates was observed (Table VII). (The activation of rat liver mitochondrial ATPase by thyroxine at 37° C. has also been observed by Maley and Johnson (1957).) The Pasteur effect was completely abolished by stilboestrol ($5 \times 10^{-5}$ M) in all experiments (Table VIII). The anaerobic glycolysis of the ascites cells was enhanced (1·5–1·8-fold) by stilboestrol only in a number of experiments with the smaller amounts of cells. In homogenates a rise of 1·4–1·5-fold in the anaerobic glycolysis was noted in the presence of stilboestrol. The oestrogen inhibited the oxidation of the ascites cells by approximately 30 per cent in the present experiments (Shacter, 1956 ; Emmelot and Bos, 1958*b*).

The observations of Barker and Lewis (1956) that thyroxine may enhance the aerobic and anaerobic glycolysis of the Ehrlich ascites carcinoma has been confirmed in the present experiments with the $S_3A$ ascites carcinoma. The results were, however, irregular, probably as a result of the decreased solubility of the hormone after slight pH changes of the medium.

*Effect of* 2: *4-dinitrophenol on the incorporation of* [1–$^{14}$C]*leucine into* $S_3A$ *ascites carcinoma protein under aerobic and anaerobic conditions*

If DNP activates the ATPases of the mitochondria of intact ascites tumour cells, it may be expected that the steady-state concentration of glycolytic ATP is lower in the presence of DNP than in its absence. This lowered ATP concentration did not inhibit the hexo- and fructokinase reactions, but might have an effect upon ATP-dependent synthetic processes such as amino acid incorporation into proteins. The following experiments (Table IX) show this to be the case. The incorporation of [1–$^{14}$C]leucine into the proteins of the ascites cells was of the same order in the presence or absence of glucose under *aerobic* conditions. Mitochondrial ATP, generated by the endogenous respiration, could thus completely satisfy the energy requirements of the amino acid incorporation processes (compare Emmelot and van Vals, 1957). Addition of DNP inhibited leucine incorporation, while addition of glucose counteracted the latter inhibition but could not restore the rate of incorporation reached in the absence of DNP. Incorporation of leucine under *anaerobic* conditions was dependent upon the presence of glucose (glycolytic ATP). With glucose present, addition of $10^{-4}$ M DNP consistently resulted in an inhibition of about 50 per cent of the anaerobic amino acid incorporation. Similar results were obtained with [1–$^{14}$C]leucine added in the range of 0·3–1·0 $\mu$mole.

It is of interest to compare the present results with those obtained with the Ehrlich ascites carcinoma. Since the anaerobic glycolysis of the Ehrlich ascites carcinoma was stimulated by DNP in our experiments (Table III), the amino acid incorporation into the protein of these cells should be depressed under the latter conditions. It has actually been reported that DNP, under anaerobic conditions and in the presence of glucose, inhibited the amino acid incorporation into the latter cells by 30 per cent (Rabinowitz, Olson and Greenberg, 1955). However, Bickis, Creaser, Quastel and Scholefield (1957) observed only a slight inhibitory effect. The latter result might have been due to the "quality" of their cells ; cells which are not in an optimal condition (e.g. as a result of repeated washings) might already show activated ATPases.

Our hypothesis demands that in those cases in which DNP does not enhance the anaerobic glycolysis, the amino acid incorporation under anaerobic conditions in the presence of glucose should not be affected either. This was found to be so in experiments carried out with slices of the mouse sarcoma UV 256 (Table IX ; Experiment 6).

### COMMENT

The present results furnish strong evidence for the supposition that the anaerobic glycolysis of ascites tumour cells may be raised by activation of the intracellular, probably mitochondrial, ATPases. The ascites cells apparently possess an enormous capacity to glycolyse, which under normal conditions cannot be attained anaerobically as a result of the relatively slow ATP-dephosphorylation. From the experiments on leucine incorporation and those conducted with the homogenates it is concluded that under anaerobic conditions the ATP-turnover increased and its steady-state concentration decreased when DNP was present.

TABLE IX.—*Effect of 2: 4-Dinitrophenol on the Incorporation of* [1–$^{14}$C] *Leucine into the Proteins of the $S_3A$ Ascites Carcinoma under Aerobic and Anaerobic Conditions in the Presence and Absence of Glucose*

Incubation in Krebs-Ringer phosphate(*) or bicarbonate(**) buffer aerobically and anaerobically (gas phase: 100% $O_2$ and 100% $N_2$; 95% $O_2$ + 5% $CO_2$ and 95% $N_2$ + 5% $CO_2$, respectively), DL-[1–$^{14}$C]-leucine (0·28 μmoles containing 0·33 μc).
Experiments 1, 2: 300 mg. wet weight of cells in 5 ml. 0·02 M phosphate buffer, 6 mg. glucose, 60 minutes.
Experiments 3, 4: 200 mg. wet weight of cells in 5 ml. 0·02 M phosphate (or $HCO_3$) buffer, 3 mg. glucose, 30 minutes.
Experiment 5: 200 mg. wet weight of cells in 6 ml. 0·03 M phosphate (or $HCO_3$) buffer, 6 mg. glucose, 30 minutes.
Experiment 6: 300 mg. wet weight of slices of sarcoma UV 256 in 3 ml. buffer, 3 mg. glucose, 30 minutes.

| Experiment No. | Glucose | DNP | Protein specific activity (c./m.) Anaerobic incubation (*) | Anaerobic incubation (**) | Aerobic incubation (*) | Aerobic incubation (**) |
|---|---|---|---|---|---|---|
| 1, 2 | — | .. | 84 | .. | 870 | .. |
| | — | $5 \cdot 10^{-5}$ | 31 | .. | 445 | .. |
| | — | $10^{-4}$ | 14 | .. | 206 | .. |
| | + | .. | 907 | .. | 945 | .. |
| | + | $5 \cdot 10^{-5}$ | 497 | .. | 537 | .. |
| | + | $10^{-4}$ | 339 | .. | 421 | .. |
| 3, 4 | — | .. | 40 | 74 | .. | .. |
| | — | $10^{-4}$ | 10 | 15 | .. | .. |
| | + | .. | 588 | 812 | .. | .. |
| | + | $10^{-4}$ | 266 | 427 | .. | .. |
| 5 | — | .. | 45 | 81 | 629 | 715 |
| | — | $10^{-4}$ | 20 | .. | 106 | 124 |
| | + | .. | 639 | 859 | 576 | 708 |
| | + | $10^{-4}$ | 326 | 428 | 315 | 371 |
| 6§ | — | .. | .. | 35 | .. | .. |
| | — | $10^{-4}$ | .. | 35 | .. | .. |
| | + | .. | .. | 314 | .. | .. |
| | + | $10^{-4}$ | .. | 322 | .. | .. |

§ Experiment carried out with slices of the mouse sarcoma UV 256.

In bone marrow cells, non-malignant and malignant lymphoblasts and slices of solid tumours no effect of DNP on the anaerobic glycolysis could be demonstrated. A variety of reasons may be responsible for this lack of effect. The glycolytic capacity may be smaller and the relative rate of ATP-dephosphorylation may be higher in the latter cells and tissues than in the ascites cells. Furthermore the rate of penetration of glucose may limit the glycolysis. In the case of tissue slices mechanical factors may be involved. Since the entrance of glucose may occur by an active transport mechanism, the number and the specific activity of the active sites in the cell membrane may also be of importance.

It has been shown that glucose penetrates into ascites tumour cells very rapidly and that the rate of glycolysis is not governed by the rate of glucose entrance (Crane, Field and Cori, 1957 ; Cori, 1956). The generally high glycolysis of ascites as well as of solid tumour cells may be due to this phenomenon, the relative differences between the two types being dependent on the unicellular or multicellular organisation (Hechter, 1957; Bloch-Frankenthal and Weinhouse, 1957).

It has been suggested (Lynen, 1958) that glucose uptake is dependent upon glycolytic ATP. The decreased aerobic glucose phosphorylation has been explained (Chance and Hess, 1956 ; Lynen, 1958) by assuming that the ATP which is generated under aerobic conditions in the mitochondria is retained and does not readily equilibrate with the cytoplasm. As a result the hexo- and fructokinase of the glycolytic pathway should be inhibited as compared with the situation under anaerobic conditions or under aerobic conditions with DNP present, in which more ATP is formed in the cytoplasm. However, the finding that the anaerobic glycolysis of the ascites cells can be markedly stimulated by increasing the dephosphorylation of glycolytic ATP, indicates that neither the uptake of glucose nor its phosphorylation is impaired by decreasing the steady-state concentration of glycolytic ATP of ascites tumour cells under anaerobic conditions.

SUMMARY

1. The effect of DNP on the aerobic and anaerobic glycolysis of ascites tumour cells, tumour slices and several types of normal and malignant cell suspensions has been studied.

DNP abolished the Pasteur effect in all cases. The anaerobic glycolysis of the Ehrlich and $S_3A$ ascites carcinomas and the T 86157 ascites lymphosarcoma was found to be stimulated by DNP ($10^{-4}$ M) about 2-fold in general, but higher increases were also observed occasionally. No effect of DNP on the anaerobic glycolysis of tumour slices (including the solid T 86157 lymphosarcoma), (non-) malignant lymphoblasts (derived from T 86157 by subculture *in vitro*) and bone marrow cells was observed. The anaerobic glycolysis of cell suspensions prepared from the solid T 86157 lymphosarcoma was enhanced by DNP under certain conditions.

2. The anaerobic glycolysis of homogenates prepared from the $S_3A$ ascites carcinoma was markedly dependent upon the concentration of $P_i$ or $P_i$ plus ADP, depending on the experimental conditions. A significant rise in the anaerobic lactate production and a drop in the $P_i$ uptake by the homogenates in the presence of DNP was observed. DNP exerted no effect when the concentration of $P_i$ (and ADP) in the medium was raised.

3. DNP stimulated ATP-dephosphorylation by homogenates and isolated mitochondria of the ascites tumour cells, but not of intact ascites tumour cells, both at 27° and 37° C.

4. Stilboestrol had a similar, though smaller, effect as DNP.

5. The energy (ATP) derived from the endogenous (fatty acid) oxidation of the ascites tumour cells was equivalent to that derived from aerobic glycolysis plus glucose oxidation, or from the anaerobic glycolysis, in sustaining amino acid incorporation into the proteins of the ascites cells. DNP inhibited amino acid incorporation into the protein of the $S_3A$ ascites carcinoma under anaerobic conditions in the presence of glucose.

6. It is concluded that under anaerobic conditions, when nearly all the $P_i$ and ADP is available to the cytoplasmic glycolysis, the rate of glycolytic-ATP generation by the ascites tumour cells is higher than the rate of ATP-dephosphorylation ; as a result the anaerobic glycolysis operates below its potential capacity. In the presence of DNP the mitochondrial ATPases are activated and the higher steady-state concentrations of $P_i$ and ADP, resulting from the splitting of glycolytic ATP by the activated mitochondrial ATPases, allow glycolysis to proceed at a higher rate than in the absence of DNP. However, since the increased ATP turnover in the presence of DNP induces a fall in the steady-state concentration of ATP, ATP-dependent anabolic processes of the ascites tumour cells, such as amino acid incorporation, are inhibited under anaerobic conditions in the presence of glucose and DNP.

Our sincere thanks are due to Dr. W. C. de Bruyn and Mr. Ch. Homburg of the Department of Experimental Cytology of this Institute for providing us with the *in vitro* cultured lymphoblasts.

### REFERENCES

ACS, G., OSTROWSKI, W. AND STRAUB, F. B.—(1954) *Acta physiol. hung.*, **6**, 261.
BARKER, S. B.—(1957) 'Methods in Enzymology' Ed. by S. P. Colowick and N. O. Kaplan. New York (Academic Press) vol. **3**, 241.
BARKER, S. B. AND LEWIS, W. J.—(1956) *Proc. Soc. exp. Biol. N.Y.*, **91**, 650.
BICKIS, I. J., CREASER, E. H., QUASTEL, J. H. AND SCHOLEFIELD, P. G.—(1957) *Nature, Lond.*, **180**, 1109.
BLOCH-FRANKENTHAL, L. AND WEINHOUSE, S.—(1957) *Cancer Res.*, **17**, 1082.
CHANCE, B. AND HESS, B.—(1956) *Ann. N.Y. Acad. Sci.*, **63**, 1008.
CLOWES, G. H. A. AND KELTCH, A. K.—(1954) *Proc. Soc. exp. Biol. N.Y.*, **86**, 629.
CORI, C. F.—(1956) 'Currents in Biochemical Research.' Ed. by D. E. Green. New York (Interscience), p. 198.
CRANE, R. K., FIELD, R. A. AND CORI, C. F.—(1957) *J. biol. Chem.*, **224**, 649.
EMMELOT, P. AND BOS, C. J.—(1955) *Rec. Trav. chim. Pays-Bas*, **74**, 1343.—(1958*a*) *Biochem. Pharmacol.*, **1**, 105.—(1958*b*) *Exp. Cell. Res.*, **14**, 132.—(1959) *Brit. J. Cancer*, **13**, 520.
*Idem*, BOS, C. J., BROMBACHER, P. J. AND HAMPE, J. F.—(1959) *Ibid.*, **13**, 348.
*Idem* AND VAN VALS, G. H.—(1957) *Ibid.*, **11**, 620.
FISKE, C. H. AND SUBBAROW, Y.—(1929) *J. biol. Chem.*, **81**, 629.
FREUND, E. AND KAMINER, G.—(1910) *Biochem. Z.*, **26**, 312.
HECHTER, O.—(1957) *Cancer Res.*, **17**, 512.
LEPAGE, G. A.—(1948) *J. biol. Chem.*, **176**, 1009.
LYNEN, F.—(1956) *Int. Congr. Biochem.*, Brussels, 1955, **3**, 294.—(1958) 'Neuere Ergebnisse aus Chemie u. Stoffwechsel der Kohlenhydrate,' Berlin (Springer Verlag), p. 155.
MALEY, G. F. AND JOHNSON, D.—(1957) *Biochem. biophys. Acta*, **26**, 522.
MEYERHOF, O. AND WILSON, J. R.—(1949) *Arch. Biochem.*, **21**, 1, 22.
RABINOWITZ, M., OLSON, M. E. AND GREENBERG, D. M.—(1955) *J. biol. Chem.*, **213**, 1.
RACKER, E.—(1956) *Ann. N.Y. Acad. Sci.*, **63**, 1017.
SHACTER, B.—(1956) *J. nat. Cancer Inst.*, **16**, 1453.
SIMON, E. W.—(1953) *Biol. Rev.*, **28**, 453.
STICKLAND, L. H.—(1956) *Biochem. J.*, **64**, 503, 515.